# Alteration in methylation level at 11β-hydroxysteroid dehydrogenase type 2 gene promoter in infants born to preeclamptic women

**DOI:** 10.1186/s12863-014-0096-5

**Published:** 2014-09-09

**Authors:** Wensheng Hu, Xiaoling Weng, Minyue Dong, Yun Liu, Wenjuan Li, Hefeng Huang

**Affiliations:** 1 Women’s Hospital, School of Medicine, Zhejiang University, 1 Xueshi Road, Hangzhou 310006, Zhejiang Province, China; 2 Institutes of Biomedical Sciences, Fudan University, 220 Handan Road, Shanghai, China; 3 Key Laboratory of Molecular Medicine, The Ministry of Education, Department of Biochemistry and Molecular Biology, Fudan University, 220 Handan Road, Shanghai, China

**Keywords:** Preeclampsia, Offspring, DNA methylation, Promoter, HSD11B2, Metabolic diseases

## Abstract

**Background:**

Preeclampsia reduces placental expression and activity of 11β-hydroxysteroid dehydrogenase type 2 (HSD11B2), leading to an increase in fetal glucocordicoids. The latter has been proposed to be associated with low birth weight and high risk of metabolic diseases in later life of the offspring. This investigation aims to delineate the alteration in methylation levels at CpG sites of HSD11B2 promoter.

**Results:**

Methylation levels of HSD9-2, HSD9-3, HSD23-2 and HSD23-3 and the mean methylation level were significantly lower in preeclampsia than in normal pregnancy (P = 0.002, 0.031, 0.047 and 0.001, respectively and P < 0.001 in mean). The mean methylation level was significantly correlated with preeclampsia after the adjustment of birth weight, maternal age, gestational age at delivery and fetal gender (r = 0.325, P < 0.001).

**Conclusions:**

Preeclampsia reduced methylation level at fetal HSD11B2 promoter. A positive correlation existed between HSD11B2 promoter methylation and preeclampsia. Our findings suggest that the methyaltion status of HSD11B2 promoter is a potentially accessible biomarker for preeclampsia. However, further studies are required to address the mechanisms of thehypomethylation at HSD11B2 promoter and the significance of the hypomethylation in the development of metabolic diseases of the fetals born to preeclamptic women.

## 1 Background

Preeclampsia is a pregnancy-specific disease characterized by de-novo development of concurrent hypertension and proteinuria and sometimes progresses into a multiorgan cluster of varying clinical features. Complicating 2-8% of pregnancies, preeclampsia remains a leading cause of maternal and perinatal mortality and morbidity [1]. Furthermore, offspring of preeclamptic pregnancies are at the high risk of metabolic diseases in late life.

Worldwide, association studies between low birth weight and the subsequent development of common cardiovascular and metabolic disorders, including hypertension, insulin resistance, type 2 diabetes and cardiovascular disease death have been observed [2]-[4]. Relatively low birth weight is one of the characteristics of preeclampsia. Intrauterine exposure to excessive glucocordicoids has been demonstrated to be one of the mechanisms leading to low birth weight. On the other hand, reduced placental expression and activity of 11β-hydroxysteroid dehydrogenase type 2 (HSD11B2) which controls cellular concentration and the transmission of cortisol from the mother to the fetus have been documented in preeclampsia, which subsequently leads to the increased fetal glucocordicoids. The later phenomenon has been considered to be one of the mechanisms programming fetal risk of metabolic diseases in adulthood.

Animal models have suggested that methylation plays a critical role in placenta development, and alterations to its methylation pattern can lead to adverse placenta morphology and birth outcome [5]. In humans, the methylation status of promoter controls the gene expression and altered methylation levels at imprinted genes have been reported to be one of the mechanisms leading to increased risk of metabolic diseases in the offspring of preeclampsia [6]. However, few researches are available yet describing the alteration in methylation status at the promoter of HSD11B2 in the offspring of preeclamptic women.

The aims of this investigation were to clarify fetal methylation status at CpG sites of the promoter of HSD11B2 in preeclampsia and normal pregnancy, and discuss the possible role of alteration in methylation at the promoter of HSD11B2 in programming of the risk of metabolic diseases in the adulthood of the offspring of preeclampsia.

## 2 Methods

### 2.1 Subjects

Forty-three women of preeclampsia (25 of mild and 18 of severe) and 78 normal pregnant women were recruited in Women’s Hospital, School of Medicine, Zhejiang University. The diagnosis of preeclampsia including severe preeclampsia and mild preeclampsia was defined as the previous study [7]-[9].

The control women had no sign of gestational complications and fetal distress and gave birth to healthy neonates of appropriate size for gestational age. All the participants are Han Chinese in origin.

The protocol of current investigation was approved by the Ethics Committee of Women’s Hospital, School of Medicine, Zhejiang University and informed consents were obtained from all the participants.

### 2.2 DNA methylation analysis

Umbilical cord blood samples were collected in Ethylene Diamine Tetraacetic Acid (EDTA)-treated tubes at delivery for subsequent DNA extraction. Genomic DNA was isolated using the QIAamp DNA Blood Mini Kit following the standard protocol (QIAGEN, Hilden, Germany) and Thermo NanoDrop2000 (Thermo, Wilmington, USA) was used to detect 260/280 nm UV absorbance ratio and concentration. Bisulfite conversion of DNA was carried out using the Epitect Bisulfite Kit (QIAGEN, Hilden, Germany).

Quantitative methylation analysis of DNA was performed using MassARRAY EpiTYPER assays (Sequenom, San Diego, CA). At the promoter of HSD11B2, primers:I-F: 5′-aggaagagagTTTTTTTGTTTTTTAGAGTTTGGGG-3′, I-R: 5′-cagtaatacgactcactatagggagaaggctAACAAAAACTAACCCAACCCTATCT-3′; II-F: 5′-aggaagagagGGTGGGTTATAAGTAATGGGAGATT-3′, II-R: 5′-cagtaatacgactcactatagggagaaggctCCACAAAACCTACCTAAAACAAAAA-3′ were designed using Epidesigner (Sequenom, San Diego, CA; http://www.epidesigner.com). Polymerase chain reaction (PCR) amplification was performed using a 5 μl reaction mixture with the following procedure: initial denaturation at 94°C for 4 min, followed by 40 cycles of 95°C for 25 sec, 58°C for 25 sec, 72°C for 70 sec and a final extension at 72°C for 7 min. The PCR products were then incubated with shrimp alkaline phosphatase (SAP, Sequenom, San Diego, CA) at 37°C for 20 min, followed by heat inactivation at 85°C for 5 min. Transcription reactions were followed according to the manufacturer’s standard protocol using T-cleavage assay kit (Sequenom, San Diego, CA). 20 μl H2O and 6 mg of Clean Resin (Sequenom, San Diego, CA) were added to the transcription products to remove bilvalent cation adducts. The samples were then sequenced on a MassARRAY analyzer (Sequenom, San Diego, CA) and analyzed with EpiTyper software (Sequenom, San Diego, CA). The amplicon comprised 6 CpG sites at the HSD11B2 DMR in Human Genome 19 assembly - Chr16: 67,462,267–67,462,481 and 67,464,230–67,464,394 (GRCh37/hg19), CpG site 1: 67,462,292; CpG site 2: 67,462,330; CpG site 3: 64,762,387; CpG site 4: 67,464,331; CpG site 5: 67,464,368; CpG site 6: 67,464,389.

### 2.3 Statistical analysis

The Kolmogorov-Smirnov tests were used to evaluate the distribution of data. Student t-tests were used for the comparison of continuous data between groups. Chi-square test was used for the analysis of categorical data. Linear mixed model analysis was used for the relationship of methylation level with the presence of preeclampsia, birth weight, maternal age, gestational age at delivery and fetal gender. Multivariate linear regression was used to evaluate the correlation of mean methylation level with birth weight, maternal age, gestational age at delivery and fetal gender. SPSS statistical package (Statistical Analysis System, Chicago, IL) was used for the data analysis. Values of P < 0.05 were considered to be statistically significant.

## 3 Results

As shown in Table 1, no significant differences between preeclamptic and control pregnancies were observed with regard to age, fetal gender. There was no significant difference in gestational age at delivery. The neonatal birth weights were significantly lower in differences in preeclampsia than normal pregnancy.

**Table 1:** Characteristics and average methylation levels of study population

	**Normal pregnancy**	**Preeclampsia**	**P value**
**TOTAL N**	78	43	
**Maternal age (y)**	28.56 ± 3.36	31.67 ± 4.68	0.356
**Gestational age (y)**	38.77 ± 1.40	36.11 ± 3.16	2.904
**Birth weigh (g)**	3287.82 ± 393.30	2626.98 ± 860.60	0.014
**HSD9_CpG_1**	0.13 ± 0.027	0.12 ± 0.031	0.279
**HSD9_CpG_2**	0.10 ± 0.019	0.09 ± 0.018	0.002
**HSD9_CpG_3**	0.41 ± 0.048	0.39 ± 0.056	0.031
**HSD9**	0.22 ± 0.026	0.21 ± 0.030	0.053
**HSD23_CpG_1**	0.16 ± 0.036	0.16 ± 0.036	0.789
**HSD23_CpG_2**	0.17 ± 0.096	0.13 ± 0.031	0.047
**HSD23_CpG_3**	0.15 ± 0.064	0.11 ± 0.048	0.001
**HSD23**	0.16 ± 0.051	0.13 ± 0.029	0.007

**Figure 1:**
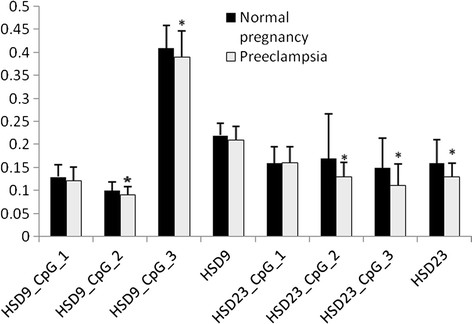
**Average methylation levels of HSD in both normal pregancy group and preeclampsia group.** There is significant differences of average methylation levels between normal pregance and preeclampsia group in HSD23_CpG2, HSD23_CpG3 and HSD23 (*P < 0.05). Data are shown as mean ± SD.

**Figure 2:**
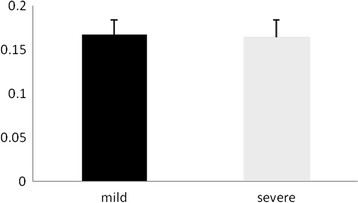
**Comparison of methylation level between mild and severe preeclampsia.** There was no significant difference in mean methylation level between mild and severe preeclampsia (P = 0.563). Data are shown as mean ± SD.

The methylation levels were detected at 6 CpG sites of HSD11B2 promoter. There were no significant differences at any of these 6 CpG sites between male and female infants in either normal pregnancy (P > 0.05 for all) (Table 2) or preeclampsia (P > 0.05 for all) (Table 1). Then, data of male and female infants were pooled. The methylation levels at sites HSD9-2, HSD9-3, HSD23-2 and HSD23-3 of HSD promoter were significantly lower in preeclampsia than in normal pregnancy (P = 0.002, 0.031, 0.047 and 0.001, respectively) (Figure 1), but the methylation levels at site HSD9-1 and HSD23-1 did not significantly differ (P = 0.279 and 0.789, respectively, Table 2) (Figure 2). The methylation levels of HSD11B2 promoter were not significantly different between mild and severe preeclampsia (P > 0.05 for all, Table 3).

**Table 2:** Average methylation levels of HSD between male and female infants

**Gender of neonater**	**Normal pregnancy**		**P value**	**Preeclampsia**		**P value**
	**Male**	**Female**		**Male**	**Female**	
**TOTAL N**	40	38		22	21	
**HSD9_CpG_1**	0.13 ± 0.032	0.13 ± 0.023	0.943	0.12 ± 0.025	0.12 ± 0.037	0.989
**HSD9_CpG_2**	0.10 ± 0.022	0.11 ± 0.016	0.358	0.09 ± 0.020	0.10 ± 0.016	0.248
**HSD9_CpG_3**	0.40 ± 0.047	0.42 ± 0.048	0.087	0.38 ± 0.063	0.40 ± 0.047	0.481
**HSD9**	0.21 ± 0.030	0.22 ± 0.020	0.341	0.20 ± 0.030	0.21 ± 0.030	0.324
**HSD23_CpG_1**	0.16 ± 0.035	0.15 ± 0.036	0.103	0.16 ± 0.034	0.15 ± 0.038	0.417
**HSD23_CpG_2**	0.16 ± 0.092	0.17 ± 0.101	0.953	0.14 ± 0.032	0.13 ± 0.032	0.498
**HSD23_CpG_3**	0.15 ± 0.060	0.15 ± 0.070	0.839	0.12 ± 0.056	0.10 ± 0.038	0.235
**HSD23**	0.16 ± 0.045	0.16 ± 0.057	0.749	0.14 ± 0.030	0.13 ± 0.030	0.190

**Table 3:** Average methylation levels of HSD in both mild and severe preeclampsia group

**Gender of neonater**	**Mild preeclampsia**	**Severe preeclampsia**	**P value**
**TOTAL N**	25	18	
**HSD9_CpG_1**	0.12 ± 0.022	0.13 ± 0.041	0.626
**HSD9_CpG_2**	0.093 ± 0.016	0.089 ± 0.022	0.505
**HSD9_CpG_3**	0.400 ± 0.044	0.38 ± 0.067	0.152
**HSD9**	0.21 ± 0.024	0.20 ± 0.037	0.690
**HSD23_CpG_1**	0.16 ± 0.034	0.15 ± 0.040	0.753
**HSD23_CpG_2**	0.13 ± 0.032	0.14 ± 0.030	0.193
**HSD23_CpG_3**	0.11 ± 0.049	0.11 ± 0.048	0.818
**HSD23**	0.13 ± 0.028	0.14 ± 0.031	0.672

The mean methylation levels at the HSD11B2 promoter were significantly different between normal pregnancy and preeclampsia (P = 0.007, Table 1). The mean methylation level was significantly lower in preeclampsia than normal pregnancy (P < 0.001). There was no significant difference in mean methylation level between mild and severe preeclampsia (P = 0.672) (Figure 2).

The mean methylation level was significantly correlated with preeclampsia after birth weight, maternal age, gestational age at delivery and fetal gender were adjusted (r = 0.325, P < 0.001), but not significantly correlated with birth weight (P = 0.165), maternal age (P = 0.778), gestational age at delivery (P = 0.064) and fetal gender (P = 0.801).

## 4 Discussion

HSD11B2 is abundantly expressed in placenta and controls intracellular concentration of cortisol as well as the transition of cortisol from the mother to the fetus. Placental expression and activity of HSD11B2 is reduced in preeclampsia with comparison to normal pregnancy [8],[9]. Diminished 11b-HSD11B22 activity which allows for more than 10-20% passage of maternal glucocorticoids leads to increased fetal blood cortisol levels in preeclampsia [10]-[12]. Glucocorticoids, such as cortisol and corticosterone control fetal and pre- and postnatal development evolving the term glucocorticoid programming. This is thought to affect the brain, the hypothalamic–pituitary axis, the blood pressure regulatory systems, the heart, glucose-insulin homeostasis and metabolism, the pancreas and fat tissue [13],[14]. Increased level of fetal glucocorticoids has been associated with low birth weight and subsequent increased risk of metabolic diseases in adulthood in humans and animal models [15]-[18].

During development, 11b-HSD11B2 is highly expressed in fetal tissues including kidney, lung, gut and notably brain [19]-[21]. However, the alteration in fetal HSD11B2 expression and activity has never been delineated yet in preeclampsia. In this investigation, we revealed for the first time the reduced fetal methylation at CpG sites of HSD11B2 promoter in preeclampsia, implying the expression of HSD11B2 is likely to be increased. Although it was reported that HSD11B2 was expressed in fetal kidney in human from early gestation, the expression and activity of HSD11B2 were determined in neither fetal kidney nor other tissues at birth. Unpublished data from our laboratory showed the expression of HSD11B2 in fetal kidney of X-induced preeclampsia model is decreased, whereas intrauterine growth restriction (IUGR) increased the methylation at CpG sites of HSD11B2 promoter in animal model. Those findings imply the complexity in the effects of pregnancy complications on fetal HSD11B2 expression and activity.

Interestingly, our present finding of a hypomethylation level at fetal HSD11B2 promoter in preeclampsia is not consistent with findings from other published studies in this area. Zhao et al. [22] found methylation levels of all studied HSD11B2 gene promoter were significantly higher in IUGR newborns. Several methodological differences may help explain such inconsistencies across the two studies. We conducted a study among 43 women of preeclampsia with healthy newborn, and we took umbilical cord blood for sample. Zhao et al. employed an experimental design with 22 IUGR newborns and used pooled sample from placenta. Moreover, CpG sites in the HSD11B2 promoter regions of our sample are not the same as those of Zhao et al. As this is an emerging area of research, more work is needed to better characterize the association of preeclampsia and HSD11B2 methylation.

Numerous mechanisms have been described to control 11b-HSD2 expression and activity. Conditions compromise HSD11B2 expression and activity including enhanced availability of pro-inflammatory cytokines (TNF-a, IL-1b and Il-6), enhanced shear stress via focal adhesions and hypoxia [23]-[26]. Hypoxia compromises the up-regulation of HSD11B2 during the differentiation of cytotrophoblasts into syncytiotrophoblasts. These conditions were present in the placenta and fetus of preeclampsia. Epigenetic modification Methylation at CpG sites of promoter is one of the epigenetic mechanisms regulating the gene expression. DNA methylation is among the best studied epigenetic modifications and the methylation of cytosine at CpG dinucleotides is an important regulatory modification of genome. Other regulatory mechanisms include transcriptional regulation and posttranscriptional modification.

Generally, the low methylation at CpG sites of promoter results in high expression of the gene. However, a partial deficit in the activity of the enzyme 11b-HSD2 was observed in essential hypertensive patients [27] and a significantly lower level messenger RNA for HSD11B2 was found in the hypertensive patients [28]. In addition, Friso et al. [29] found that elevated HSD11B2 promoter methylation was associated with hypertension developing in glucocorticoid-treated patients and essential hypertension. Taken these observations together with our findings, it is implied that hypomethylation status at the promoter of HSD11B2 may not be among the mechanisms linking intrauterine exposure to maternal preeclampsia and high risk of metabolic diseases in adulthood.

Abnormal hypermethylated or hypomethylated genes can serve as biomarkers for clinical use in early detection, classification, and prediction of treatment response. In our study, we detected the alteration in methylation status at the promoter of HSD11B2 in the offspring of preeclamptic women. We finally found that methylation levels of HSD11B2 promoter were significantly lower in preeclampsia than normal pregnancy. The positive relationship between HSD11B2 promoter methylation and infants born to preeclamptic women with the hypomethylated HSD11B2 may be a potential marker for preeclampsia. However, further studies are required to address the mechanisms leading fetal hypomethylation at HSD11B2 promoter and the significance of the hypomethylation in the development of metabolic diseases.

## 5 Conclusions

In conclusion, our study suggests that the decreased methylation at the promoter of HSD in the fetuses of preeclamptic women could be a potentially accessible biomarker for preeclampsia women. Further studies are required to explain the mechanisms of hypomethylation at HSD11B2 promoter in infants born to preeclamptic women.

## Competing interests

The authors declare that they have no competing interests.

## Authors’ contributions

WH conceived of the study, participated in the design of the study, performed statistical analyses, and drafted the manuscript. XW carried out the genome-wide genotyping, targeted sequencing, quality control, performed statistical analysis, and drafted the manuscript. MD performed statistical analysis and aided in the drafting of the manuscript. YL, and WL participated in the design of the study and drafting the manuscript. HH conceived of the study, participated in its design and coordination, and helped to draft the manuscript. All authors read and approved the final manuscript.
